# Exploring the use of health and wellbeing measures during pregnancy and the first year following birth in women living with pre-existing long-term conditions: qualitative interviews with women and healthcare professionals

**DOI:** 10.1186/s12913-021-06615-w

**Published:** 2021-06-24

**Authors:** Laura Kelly, Jennifer J. Kurinczuk, Oliver Rivero-Arias, Ray Fitzpatrick, Elizabeth Gibbons, Fiona Alderdice

**Affiliations:** 1 Health Services Research Unit, Nuffield Department of Population Health, University of Oxford, Oxford, UK; 2 Harris Manchester College, Oxford, UK; 3 National Perinatal Epidemiology Unit, Nuffield Department of Population Health, University of Oxford, Oxford, UK; 4 Clinical Outcomes Solutions, Oxford, UK

**Keywords:** Maternity care, Qualitative interviews, Patient-reported outcomes, Questionnaire, Chronic conditions, Pregnancy, Postpartum

## Abstract

**Background:**

One way in which care for pregnant and postpartum women living with long-term health conditions (LTCs) may be improved is through the adoption of standardised measures to provide evidence of health outcomes and wellbeing from the woman’s perspective.

**Aim:**

The study explores the views of pregnant and postpartum women living with LTCs, and healthcare professionals to better understand the potential value of using standardised health and wellbeing measures within this patient population.

**Methods:**

Qualitative semi-structured telephone interviews were conducted to explore the perceived value of using measures with pregnant and postpartum women living with LTCs within maternity services. Participants were asked to provide feedback on three exemplar measures: the Long Term Conditions Questionnaire, the Wellbeing in Pregnancy Questionnaire and the EuroQol EQ-5D-5L instrument. Thematic analysis was used in the analysis of the transcripts.

**Results:**

Eleven women and 11 healthcare professionals took part in semi-structured interviews. Analysis identified five themes as relevant to the use of measures within maternity services: 1) Improving care, 2) Assessing outcomes, 3) Interpretation and application of data, 4) Engagement challenges and implementation and, 5) Women and healthcare professionals alignment.

**Conclusions:**

Despite varying prior experience and expressing some questions about implementation, respondents were cautiously positive about the use of standardised health and wellbeing measures. Their use offers the opportunity for both affected women and healthcare professionals caring for them to collectively identify and assess important areas of unmet needs and improve outcomes. Incorporating the perspectives of women with LTC’s will help bring awareness to elements of women centred care which health services may seek to address.

**Supplementary Information:**

The online version contains supplementary material available at 10.1186/s12913-021-06615-w.

## Introduction

The prevalence of long-term conditions (LTCs) in women of reproductive age is increasing [1, 2]. In 2018, 9.6% of women in the UK reported having a pre-existing LTC which complicated their pregnancy [3]. The most common conditions reported included high blood pressure (4.3%), diabetes (3.2%), back problems (2.8%) and thyroid problems (1.2%). Complex pregnancies due to pre-existing health problems can result in adverse pregnancy related outcomes and impact on women’s experiences of pregnancy, childbirth and early parenting [4–7]. Women with LTCs are also less likely to initiate or maintain breast-feeding when compared to their counterparts who are not living with LTCs [8].

During the years 2015–2017, 67% of maternal deaths in the UK were women with pre-existing medical problems with cardiac disease, epilepsy and stroke documented as the highest indirect contributors [3]. Many women living with LTCs are therefore considered ‘high-risk’ in maternity care and may require management across a range of services including specialist clinics, day assessment units and hospital admission. Confidential Enquiry Reports however suggest that these women experience fragmented care and inadequate communication between different healthcare professionals and specialities, which puts their health and wellbeing at risk [9].

High quality and effective pregnancy and postpartum care for women living with pre-existing LTCs has the potential to offer lifelong benefits for a woman, her baby and wider family, with indirect benefits extending to society. Evidence incorporating women’s perspectives is required to assess support unmet needs, experiences of care and important outcomes for these high-risk groups [10, 11]. Including women’s perspectives involves asking questions they see as relevant about their health and care during antenatal and postpartum care and requires us to understand how they report on health and wellbeing during this time.

Patient-reported outcome measures (PROMs) are instruments used to gain insights from the patients’ perspective (without interpretation by a clinician or anyone else) regarding the status of their health condition [12]. PROMs can measure a wide range of constructs such as health status, quality of life, wellbeing, treatment satisfaction, symptoms and functional status [13, 14]. Standardised health and wellbeing measures may offer an opportunity for women to report health and help enable healthcare professionals to identify unmet needs, identify variations across patient condition groups and the broader maternity population as a whole. Whilst these standardised measures have historically been used to compare outcomes in the clinical trial context, patient reported data are increasingly used in clinical practice to evaluate health and wellbeing from the patient’s perspective [15–17].

The use of health and wellbeing measures with women during pregnancy and postpartum appears to be limited but their potential value in this field is being recognised [18–20]. A recent review has indicated that the use of PROMs in pregnancy and childbirth have largely been in the context of three main areas: 1) in assessing the impact of disease or complication (for example, postpartum haemorrhage); 2) to assess impacts of specific care interventions or, 3) in the assessment of a new PROM for maternity care [21]. Very few published PROMs exist for use in pregnant or postpartum women, nor have condition-specific measures been tested or validated for use in this cohort of women [18, 22]. Measures tend to concentrate on pregnancy mediated health complications, for example, gestational diabetes [18]. There are therefore missed opportunities to generate patient driven data which can support clinical decision-making, patient choice, target resources and assess outcomes, such as wellbeing or quality of life in women with pre-existing LTCs [21, 23].

Our understanding of the value which healthcare professionals place on the use of health and wellbeing measures in the maternity setting is also limited. Research has been carried out to understand the perceived value of using such measures with patients in a variety of contexts, such as living well with long-term conditions or assessing the integration of health and social care [24, 25]. No studies however have determined how healthcare professionals working within maternity services might value their use in services.

We aimed to explore the views of pregnant and postpartum women living with LTCs, and the views of healthcare professionals to better understand the potential value of using health and wellbeing measures within this patient population. To gain an insight into the value of measures within the maternity setting, we presented three exemplar instruments which provided context to participants. The first measure, the Long Term Conditions Questionnaire (LTCQ), has been developed to capture the impact of living with physical or mental LTCs [26]. To date, the LTCQ has been evaluated in a large and diverse sample of health and social care users. Although younger people were included in the study, a significant proportion was older. Further evaluation is needed to establish its acceptability for use in younger population groups who are living with LTCs. The second measure, the Wellbeing in Pregnancy Questionnaire (WiP), assesses pregnancy related positive and negative affect and life satisfaction [27]. Preliminary development and analysis of the WiP was conducted using data collected from pregnant women who largely reported good health. Further assessment is required to explore its use in women living with LTCs. The third measure, the EuroQol EQ-5D-5L, is a widely used health-related quality of life measure commonly used in cost-effectiveness analysis [28]. This measure has been widely used in over 80 clinical areas including many LTCs [29] but the evidence of acceptability for use with pregnant women with LTCs is virtually non-existent.

## Methods

Qualitative semi-structured phone interviews were conducted with women and healthcare professionals to explore the value of health and wellbeing measures for use with pregnant and postpartum women living with one or more pre-existing LTC. Interviews also explored the appropriateness of three specific exemplar measures for use within this population. Ethical approval was granted by the University of Oxford’s Medical Sciences Interdivisional Research Ethics Committee (Reference Number: R61498/RE001). All participants provided written informed consent via a secure online consent form prior to taking part.

### Participants

Participants included were pregnant and postpartum women living with one or more LTC, and healthcare professionals working within maternity services. Women were required to be living in the UK, be at least 18 years old and either currently pregnant or had given birth within the past year. We sought women to represent a range of (self-reported) conditions. Targeted conditions included asthma, diabetes, epilepsy, heart problems, gastrointestinal problems, mental health problems, back pain and thyroid problems. These represented conditions which are commonly reported problems affecting pregnancy and which represented high-risk conditions which can result in adverse pregnancy related outcomes for both mother and baby [3–7, 9, 30]. Note, that recruitment was not limited to the listed conditions. Healthcare professionals were required to have experience of caring for pregnant women in connection to their LTC (such as, GPs, midwives, obstetricians and health visitors). We sought representation of at least two healthcare professionals from each identified professional discipline.

### Recruitment

Pregnant and postpartum women living with LTCs were recruited through a range of stakeholder groups representing pregnant and postpartum women. These included NCT (formerly the National Childbirth Trust), Mumsnet, Netmums, Mums Like Us and a Baby Café. Recruitment also took place through UK national charities for specific LTCs including, Epilepsy Action, Mc Pin Foundation, Diabetes UK and Diabetes Support Forum UK. Healthcare professionals were recruited through existing research networks, and through relevant representative bodies such as the Royal College of Midwives and the Royal College of Obstetricians and Gynaecologists. For both women living with LTCs and healthcare professionals, further recruitment took place through participant peer referrals where a participant provided peers with the authors email address so they could make contact to find out further information. Women living with LTCs received a £30 voucher for participating.

### Procedure

Recruitment advertisements for women living with LTCs were posted on charity websites and/or on their internet message boards or social media platforms. Healthcare professionals were emailed directly with a participant information sheet attached, providing full details of the project.

### Data collection

The interview topic guide (see Supplementary file 1) aimed at women with LTCs included questions which focused on their experiences of maternity care, impacts of their LTCs during pregnancy and the postpartum period, the potential value of health and wellbeing measures in clinical care and research contexts. The interview topic guide (see Supplementary file 1) aimed at healthcare professionals focused on their current professional role, experiences of caring for pregnant and postpartum women living with LTCs, and potential value of health and wellbeing measures in clinical care or research. All participants were shown three exemplar measures which reflected different aspects of health and wellbeing. As outlined previously, the measures included the Long-term Conditions Questionnaire (LTCQ), the Wellbeing in Pregnancy questionnaire (WiP) and the EQ-5D-5L. Participants were consulted on the relevance of each questionnaire’s content in addition to their perceived usefulness in relation to pregnancy. Participant responses were followed up with probes. Interviews were recorded, transcribed verbatim by a professional transcriber and, anonymised and checked for accuracy by the research team on their return.

### Analysis

Thematic analysis was carried out using QSR NVIVO 11 which facilitates management of the data [31]. Analysis consisted of four steps: (1) familiarization with the interview data and devising a coding framework; (2) identification of a thematic framework which allowed for emerging issues and concepts to be organised; (3) indexing transcripts according to the thematic framework and; (4) describing and summarising the themes identified [32]. Codes were selected using a deductive approach, where codes were preselected based on previous literature [17, 24, 33]; however, analysis allowed for an inductive and open coding where emerging concepts were elicited [34]. One coding framework was devised for use with all interview transcripts as it was anticipated that many topics discussed would be of relevance for both the women interviewed and the healthcare professionals. In addition, participant reflections on the three exemplar questionnaires were analysed whereby thoughts on individual items were identified within NVIVO and collated in Microsoft Excel sheets to assess each questionnaire. Finally, to retain anonymity throughout the text, some healthcare professionals were grouped at a high-level occupational discipline, i.e. the obstetricians and the obstetric physician were grouped under ‘Obstetrician/Obstetric Physician’.

## Results

A total of 11 women with pre-existing LTCs and 11 healthcare professionals took part in the semi-structured interviews. Women were a mean age of 32.9 years (SD 3.8, range 26–38 years). At the time of interviewing, five women were pregnant. Nine women reported that they had previously given birth, with six women reporting giving birth within the previous year. Six women reported living with more than one LTC. See Table 1 for the range of conditions reported.

**Table 1:** Participant characteristics

**Women with LTC**	***N*** **= 11**
**Age, Mean years (SD)**	32.9 (3.8)
**Condition, N**^**a**^	
Asthma	3
Diabetes (Type 1)	2
Heart problems (Defective mitral valve)	1
Stomach or colon problems (Ulcerative colitis)	1
Depression, anxiety	1
Back pain	1
Spinal condition (Diastematomyelia)	1
Hypothyroidism	3
Chronic rhinitis	1
Multiple Sclerosis	1
Endometriosis	1
Pernicious anaemia	1
**Living children, N**	
0	2
1	6
2	2
3	1
**Currently pregnant, N**	5
**Healthcare professional**	***N*** **= 11**
**Role, N**	
Obstetric physician	1
GP	1
Obstetrician	3
Midwife	3
Psychiatrist	1
Health Visitor	2

*GP* general practitioner, *NHS* National Health Service

^a^Six participants reported having more than one condition

Healthcare professional’s roles included a GP with a special interest in perinatal health and high-risk pregnancies, a specialist midwife for hypertension and renal disease, a diabetes specialist midwife, a midwife and infant feeding co-ordinator, three obstetricians, an obstetric physician (grouped under ‘Obstetrician/Obstetric Physician’ hereafter to protect anonymity), a consultant perinatal psychiatrist and two health visitors. All healthcare professionals reported extensive experience of working with pregnant and/or postpartum women living with pre-existing LTCs. Experience of using health or wellbeing measures varied among the sample. Six healthcare professionals reported no experience of using validated tools in clinical practice, whilst others reported some use of screening instruments. Screening tools reported included use of the Whooley questions for depression, the Patient Health Questionnaire (PHQ-2 and PHQ-9), the Edinburgh Postnatal Depression Scale (EPDS) and, the Generalized Anxiety Disorder scale (GAD-2 and GAD-7). The use of the Strengths and Difficulties Questionnaire for children and the UNICEF Breastfeeding Assessment reported by one health visitor, and one obstetrician/obstetric physician reported the use of her own ‘tailored’ questionnaire for her debrief clinic’s for women who experienced traumatic births.

### Themes

Analysis identified five main themes: 1) Improving care, 2) Assessing outcomes, 3) Interpretation and application of data, 4) Engagement challenges and implementation and, 5) Women and healthcare professional alignment.

#### Improving care

Women and healthcare professionals recognised the value of using health and wellbeing measures to improve care. Women welcomed the use of these measures within maternity care and thought they would be useful tools to help support them during consultations. Seven women felt that completing the exemplar questions shown to them during their interview would help them to remember to ask relevant questions and act as a reminder to bring up important topics of concern during a consultation. One woman living with multiple sclerosis said:

*…from a patient’s point of view you can’t remember to say everything you want to say… They’re [questionnaires] little like triggers so that you …remember, or say things that aren’t necessarily in the forefront of your mind because something more important is happening.* ***W11, Multiple sclerosis***

Women living with LTCs explained that they often have complex needs as they were dealing with changes in their body due to pregnancy and the impact of childbirth. Women not only welcomed the opportunity to be involved in their own care, through the use of questionnaires; but also discussed the desire to talk about their personal health in an environment where they felt often that the emphasis was predominantly on the health of their baby. Women felt that completing the exemplar measures gave them the opportunity to open conversations on how they were feeling about their own health and wellbeing. This extended to the postpartum period. Women living with conditions requiring frequent contact with services during pregnancy, such as, diabetes and ulcerative colitis, found it difficult to access specialist knowledge about their LTC after the birth. Postnatal care predominantly consisted of interactions with community midwives or health visitors. In this context one woman explained:

…the thing that I found difficult, even waiting a couple of weeks [after birth] … to see a consultant or somebody at the diabetes care team. If these [LTCQ] questions were asked earlier on then little issues that I might have been having, or my blood sugars, could have been resolved quicker….obviously, the midwives do ask questions …[they were] about the baby … the community midwives don’t have the understanding about the diabetes. **W2, Diabetes (Type 1) and hypothyroidism**

Healthcare professionals looked positively upon health and wellbeing measures as useful tools with which to facilitate dialogue, open up and identify areas of focus during consultations. Five healthcare professionals commented that using these measures could identify important support needs that could otherwise remain unknown. One obstetrician/obstetric physician articulated how the WiP could enable healthcare professionals to develop appropriate lines of enquiry:

*[The WiP] …could give you openings into areas that you need to address …‘which area is it that she's particularly worried about?’…make them explore areas that you wouldn’t explore if you were just asking an open question..* ***SH3, Obstetrician/Obstetric Physician***

In addition to improving communication between healthcare professionals and women using maternity services, some healthcare professionals recognised the value of health and wellbeing measures within multidisciplinary settings. Using standardised measures was seen as an opportunity to share information between cross-functional colleagues to provide collective insight into a woman’s support needs. One health visitor said:

*...if we’re then highlighting concerns to other professionals it is really useful to have a standardised tool that we can all relate to.* ***SH11, Health visitor***

Throughout the interviews, there was a consensus that any added benefit of using health and wellbeing measures in a clinical care context needed to have a clear purpose and that they should not become an administrative box ticking exercise.

#### Assessing outcomes

Health professionals discussed the use of health and wellbeing measures for assessing outcomes. Using measures to evaluate outcomes was viewed as particularly important in the context of assessing interventions where traditional endpoints were either difficult or inappropriate to measure. Three obstetrician/obstetric physicians gave the example of their potential use for capturing the outcomes of pre-pregnancy counselling services for women living with pre-existing LTCs. One commented:

*…pregnancy counselling is very expensive….You can count deaths, you can count morbidity but linking that to pre-pregnancy counselling and ... avoiding anxiety in pregnancy because you’ve counselled the woman before, is impossible with our current tools. And so, if you were to come up with a tool that allowed me, or us, to measure; get a handle on how information is power and preparation helps the woman deal with … an adverse pregnancy outcome, because she's been counselled …measuring that is what I would like to be able to do through some form of questionnaire.* ***SH1, Obstetrician/Obstetric Physician***

Health visitors also discussed the difficulties of capturing outcomes of their services. One health visitor felt that health and wellbeing measures would be useful to monitor outcomes and measure the impact of their role, whilst the second highlighted the difficulties of capturing the complexity of their role:

*…it’s always difficult, I think particularly with mental health, to identify what it is that our role has done, what impact has our role made on clients? So actually, it is useful to have … that comparison.* ***SH11, Health visitor***

*…if you're trying to highlight the skill and the expertise and the complexity that health visitors are working with all the time, then actually I could see that … these might be something that might be useful. Um I think more around demonstrating that complexity that we work with on a daily basis that’s often not recognised.* ***SH9, Health visitor***

The women interviewed demonstrated more interest in completing measures as part of their individual care rather than to assess outcomes of services or research interventions. All indicated however that they would be happy to complete health and wellbeing measures if they were to inform research or monitor outcomes of a service.

*Yeah, I just think that as long as you warn people that it’s not going to be used for… their own care…then I would be totally fine with…having these [questions] asked and answering them.* ***W8, Asthma, Heart problems, Pernicious anaemia***

#### The use and interpretation of data

Healthcare professionals indicated that they would require information on how to interpret scores for a specific measure for it to be informative. Two obstetrician/obstetric physicians and one GP advised that they would require baseline data and an indication of threshold scores for making referrals. One obstetrician said:

*I think, before you could put them [measures] into day-to-day practice, you need the baseline data of what to expect and resources to know when to actually say, 'Well this is a woman who's really struggling from a mental health perspective with a pregnancy; she needs some support… So, is it normal that someone gets much more worried about the birth of a baby as she approaches that time; or is that something that people worry about all the way through the pregnancy?'* ***SH3, Obstetrician/Obstetric Physician***

Interviewees discussed the importance of having clear referral pathways if measures were used in health services. Some healthcare professionals expressed concern regarding the lack of referral options, for example, in cases where the need for mental health support became apparent. Others worried that it was not feasible to address certain support needs within a relatively short timeframe. One obstetrician/obstetric physician said:

*…one has to be a bit realistic of what…obstetric services can sort out in effectively six or nine months….... you could signpost... if you delve into things for people you need to be able to offer a support or information or use it in their management plan…. it’s something the GP looking after someone with long term conditions should be assessing…. Optimise their health before [pregnancy] … I feel very strongly about GPs moving out of obstetric care, but they still have a huge responsibility in…getting people optimum for pregnancy* ***SH5, Obstetrician/Obstetric Physician***

Two women living with LTCs felt that health services should use health and wellbeing data to inform new practices. Women could then be reassured that resulting interventions were based on evidence and feedback from women who had used services in the past. One woman living with Type 1 diabetes and hypothyroidism said:

*…[A HCP can]… say, 'We've populated this [questionnaire data] across a thousand women in the North East region and this is what the outcomes are …what we're going to try this time is as a result of this feedback.* ***W2, Diabetes (Type 1) and hypothyroidism***

In cases where measures are to inform an individual woman’s care, there was an overriding consensus among all participants that questions should be interpreted on an individual item basis so that important responses would not be missed. This is reflected in the thoughts of a diabetes specialist midwife and by a woman living with hypothyroidism:

*For me, if someone said they felt lonely, that would be a red flag because in my mind that could lead to so many other issues when you’ve got someone that’s about to become a new mother, and yet at the same time they could feel completely safe in their home… everything else might be really quite alright, but if they felt really isolated and lonely who knows where that might lead to? So, yeah, I would want to have the individual responses and not just an overall score.* ***SH10, Diabetes specialist midwife***

*I think the data collection points are great for service improvement and whatnot, but it's the conversation that’s going to make me as a [slight pause] as a person feel better. You know, you can score it all you like, but that doesn’t mean anything [to me].* ***W10, Hypothyroidism***

A further suggested use of data was to monitor individual change over time. One consultant perinatal psychiatrist discussed the value of following scores on a specific measure over time. From a pregnant woman’s perspective, one woman discussed potential feelings of encouragement when seeing improvement in scores, whilst also highlighting areas for further management:

*…I think it's quite a good way of showing an outcome….it's quite a nice thing to be able to show the patients that this is what you said; this is how you think things are progressing…Certainly finding a scale which resonates with our patient is really useful.* ***SH6, Consultant perinatal psychiatrist***

*I think to be able to access them if I'm sort of sitting and self-analysing, to be able to access them and say, '… Look how different I'm responding to that question!,' and particularly if it's one that was quite upsetting perhaps, to have a marker of improvement would be very encouraging. But likewise, to be able to see if something's declining so that if no-one else is flagging it up you can sort of... you can perhaps flag it yourself if it's something you're keeping an eye on.* ***W1, Type 1 diabetes***

#### Challenges with engagement and implementation

Amongst the healthcare professionals within the sample, health visitors tended to discuss health and wellbeing questionnaires with most familiarity. The two health visitors interviewed described using standardised questionnaires as communication instruments with women in their care and were both receptive towards their use. It was, however, notable that the use of health and wellbeing measures within maternity care services was not common practice and some concerns were expressed when considering their adoption.

The obstetrician/obstetric physicians and GP within the sample considered their clinical training as key to identifying problems. It was within this context that they questioned the ‘added value’ of using health and wellbeing measures during their consultation as they already had protocols in place for gathering information to make assessments:

*... I'm struggling slightly to see how, if I take a good health history, and ask the right questions with the patient, why would I need this?...But that said, you know, doctors are fallible; midwives are fallible; we don’t always ask all the questions; we miss stuff.* ***SH1, Obstetrician/Obstetric Physician***

Midwives were receptive towards using health and wellbeing measures within maternity care. Although generally positive towards their use however, one diabetes specialist remarked that some questions within the exemplar measures felt unnatural or repetitive as she had very frequent consultations with her patients and was therefore well acquainted with the women’s support needs. The infant feeding co-ordinator thought the measures would be useful for during a woman’s care; however, felt they were somewhat limited for use within her particular role as a breastfeeding consultant. In this setting, she felt an unstructured consultation worked best.

Two healthcare professionals and one woman cautioned that it was important to be mindful of the context in which specific measures are administered to ensure accuracy of responses. For example, women may find it uncomfortable to respond to questionnaire items regarding sensitive topics or satisfaction with care received. One woman living with a LTC commented:

*I'm just a little bit cautious about questionnaires that you complete that are just fed back instantly, um and then alters your care.* ***W6, Ulcerative colitis***

Whilst the perinatal psychiatrist felt the exemplar measures were best asked in a face-to-face context, some concerns were raised over the accuracy of responses for some sensitive questions:

*…unless you sort of changed the wording a little bit of that, that wellbeing in pregnancy one, I think you need to... they need to have permission to say I don’t like my baby which is a really, really hard thing to say.* ***SH6, Consultant perinatal psychiatrist***

Whilst it was acknowledged completing measures within maternity services could potentially require some additional resources, most interviewees did not raise this as a critical issue. Women living with LTCs felt that they would be a useful addition to their care and potentially allow resources to be focused on where they are most needed. Two women living with LTCs advised:

*… I personally feel like they [the LTCQ and WiP] should be used [with] anybody who gets pregnant, and is … in and out of hospital with a long-term condition. I feel like any time that they see their obstetrician, or see a midwife, the midwife or whoever should at least talk to them or offer them these questionnaires to try and get a sense of how the pregnant lady with the long-term health condition is feeling … Because healthcare professionals don’t really have a great deal of time. So if they had a questionnaire like that to read off then they could maybe go into it a little bit more and say, ‘Okay, what’s this problem about?’* ***W4, Spinal condition: Diastematomyelia***

*…you could upload them to your maternity notes and you complete them and then a midwife gives you a call or something to go through them. I don’t think you necessarily need to sit with someone to do it.* ***W12, Endometriosis and underactive thyroid***

#### Women and healthcare professional alignment

Participants expressed varied views on the relevance of some individual items within the exemplar measures. On some domains, it was evident that there was some misalignment between important elements to include for women in this cohort. Differences arose between healthcare professionals and the patient perspective, but also between healthcare professional’s roles. For example, reflecting on an item in the LTCQ about loneliness, two obstetrician/obstetric physicians agreed that it was not critical for inclusion as it was more relevant to older frail people than the women they see or it presented too much overlap with other items that focused on emotional wellbeing. In contrast, a diabetes specialist midwife thought it one of the most relevant questions for her patients within the measure as many women are unaware that others are going through similar experiences. One woman described the importance of including items on the emotional aspects of health and, in particular, the item regarding loneliness:

*I do think that that’s [loneliness] a good question, and I don’t think that it’s something that you get asked a lot. In all my pregnancies that’s one thing that I noticed, is that … The doctors are very concerned that I have bed rest, um and that my heart is not going to give me problems and that I’m not going to all of a sudden keel over and die, but no one is actually concerned about the fact that the whole thing is causing a huge amount of stress … suddenly you feel like you’re losing your mind. … even when you address it I get the feeling that those health professionals are a bit like, ‘Yes, we know that’s important, but right now that is not as important as the rest,’ and you are left a bit like, ‘Well, for me it is.’ …I think …the ‘felt lonely’, the ‘felt bothered’, even the ‘felt more dependent on others’ and ‘just feeling safe’, I think that those point to how little you are coping with it.* ***W8, Asthma, Heart problems Pernicious anaemia***

A further example of contrasting opinions regarding items was the WiP item ‘*I am happy with how I look in pregnancy*’. Whilst some healthcare professionals were sceptical of its relevance to caring for women with LTCs, a midwife welcomed its inclusion as a way of opening up relevant topics and a woman interviewed felt it resonated deeply with her feelings when using services:

*Happy how I look in pregnancy? What are you going to do about that?... you can take that out.* ***SH5, Obstetrician/Obstetric Physician***

*When you're looking after someone in pregnancy you often think about their emotional wellbeing and their physical wellbeing, but I don’t think I’ve ever asked the question, ‘How are you feeling about your body changing?’ There certainly are some women who that might be a trigger for in terms of feeling low in mood or unhappy about their pregnancy. It’s something I’ve probably never thought about before, but perhaps it is quite a useful question.* ***SH8, Specialist midwife for hypertension and renal disease***

*I’ve been unhappy with how I look during pregnancy, but it’s not something that I’ve spoken about …I’ve been concerned just even going to see a consultant where you have to … have a scan as well, where you have to kind of undress slightly, and my hips and everything, everything’s all a strange shape and I feel like I’m having to apologise to those people about my body…As somebody with a long-term condition I am kind of happy that that question is in the questionnaire.* ***W4, Spinal condition (Diastematomyelia)***

Both women and healthcare professionals identified the EQ-5D-5L as the most general measure of the three discussed. Whilst the EQ-5D-5L was considered simple, quick and easy to complete, it was generally recognised as primarily useful for providing a high-level overview of health. One woman interviewed reflected on the measures value:

*…even though it's [EQ-5D-5L] so generic, you can actually get quite specific information and you can get a good idea of someone’s health status at that point…the nuances are not there, you don’t know why they're having a problem walking, why they can't take care of themselves, but it gives you a very good overview of their health.* ***W6, Ulcerative colitis***

Consistent with previous research [35], a health professional working with women with severe mental health conditions expressed concern regarding the use of the EQ-5D-5L and how patient responses should be interpreted:

*…our patients really struggle with these sorts of questions... in terms of where do they place themselves and what's been distorted by how they're feeling. So, if they're severely depressed then they think ‘I can't do anything’ and give you very opposite answers … if you were severely anxious, you're sort of almost over confident…and sort of say, 'Well, I can do all these things but ... I'm just feeling really anxious all the way through, and in actual fact ... they almost over estimate what they can do….And so, things like self-care, then they're sort of like, 'Well, I can do it,' but the reality is that she might just take them a very long time to do it... So, if you’ve got OCD they're having to count every time they touch a tap and all these sort of things, then they're anxious….from my point of view actually, that’s a very severe problem.* ***SH6, Consultant perinatal psychiatrist***

## Discussion

Healthcare services are under increasing pressure to support pregnant and postpartum women who are living with LTCs. These women need to be supported to improve wellbeing, prevent further health-related complications and ensure the best outcomes for their baby. Health and wellbeing measures may be useful resources for both women using services and those working within health services to identify needs and assess outcomes. Limited investigations into the use of such measures within this cohort of women have been reported. This study aimed to explore the views of pregnant and postpartum women living with LTCs, and the views of healthcare professionals regarding the value of using health and wellbeing measures within this context.

Semi-structured interviews with pregnant and postpartum women living with LTCs and healthcare professionals indicated that participants felt the use of measures could, first, improve care through improved communication and identification of support needs and, second, evaluate service or research outcomes. Improving communication and having a mechanism to incorporate the woman’s perspective in her care was valuable, particularly in light of some misalignment regarding unmet needs. Whilst it was to be expected that different healthcare professionals would prioritise aspects of care differently depending on their role, giving women the opportunity to highlight important areas of concern from their perspective in a structured manner may help address worries or concerns, or identify particular support needs. Previous research supports the importance of including the patients perspective through the use of health and wellbeing measures so that healthcare professionals fully understand the impact a condition may have on daily life, the severity with which a patient may experience symptoms, add value to the clinical encounter and, to help develop treatment plans [18, 36–39].

In the diverse sample of healthcare professionals interviewed whose responsibilities spanned the complete maternity pathway for women living with LTCs, it was evident that there was a sense of unfamiliarity with the use of measures. This was somewhat expected as, whilst health and wellbeing measures are established in some therapeutic areas, it is not uncommon for there to be a gap between health services research and the awareness of available measures among front line healthcare or social care service providers [25, 40]. When shown three exemplar measures, there was however a recognition of their potential value in assessing difficult to capture outcomes of care, for example, outcomes of pre-conception counselling services. Whilst previous research has shown benefits of pre-conception counselling, identifying effective elements of counselling is an important next step in the development of a strong evidence base for pre-conception services for women living with LTCs [2, 4]. Women interviewed also highlighted the potential use of measures in the year following birth to highlight care and support needs, particularly where they are no longer under specialist care. Identifying specific postnatal care and support needs of women with diabetes has also been identified by patients and healthcare professionals in a James Lind Alliance priority setting partnership recognising key research priorities diabetes in pregnancy [41].

This research also identified areas for future investigations. As with findings in other areas of health care [24, 42], participants indicated some uncertainty in how they should interpret patient responses and group data. As a priority, establishing the validity of the LTCQ, the WiP and the EQ-5D-5L using an adequate sample size for use within this group of women will advance our knowledge and improve interpretation of scores. Data should be analysed to assess whether the internal structures of the measures are consistent with the underlying constructs upon which the measures are based, and to evaluate properties such as potential floor and ceiling effects or score distributions [43]. Further exploration is needed to understand how scores may differ across LTC groups and how important variations or changes in scores may be interpreted.

It was clear that participants were cautiously positive towards the use of health and wellbeing measures with pregnant and postpartum women living with LTCs. The use of health and wellbeing measures within maternity services however is in its infancy and further engagement would require buy-in from healthcare professionals and an understanding that these measures are constructed in a robust scientific way. For example, it was noted that measures were sometimes referred to by healthcare professionals as ‘qualitative’ data suggesting they were not viewed as scientific instruments. In this respect, it may be informative to take key learnings from other health services where these measures have been adopted to demonstrate potential uses. For example, experiences of integrating PROMs in routine practice to enhance clinical care in oncology services [16].

Some limitations of this research must be acknowledged which are largely related to the transferability of the findings. Whilst we endeavoured to include a wide representation of women with LTCs, it is acknowledged that the sample does not include representation from all high-risk groups in maternity care, for example, women living with epilepsy. It will be important to make substantial efforts to recruit women living with epilepsy in any future research determining acceptability of health and wellbeing measures.

## Conclusion

High quality and effective perinatal care for women living with pre-existing LTCs has the potential to offer lifelong benefits for a woman, her baby and wider family. The use of standardised health and wellbeing measures offers the opportunity for both healthcare professionals and women in their care to collectively identify and assess important areas of unmet needs and improve outcomes. Incorporating the perspectives of women with LTC’s will help to bring awareness to elements of women centred care which health services may seek to address.

To ensure existing measures are both valid and reliable for use among perinatal women living with LTCs, it is imperative that they are assessed for acceptability among a larger sample encompassing a wider group of LTCs to assess psychometric properties within this population group.

## Supplementary Information

**Additional file 1.**

## Data Availability

The datasets generated and analysed during the current study are not publicly available due to ethical concerns regarding the possibility to fully anonymise data but are available from the corresponding author on reasonable request.
